# Adverse events in the treatment of MDR-TB patients within and outside the NTP in Pham Ngoc Thach hospital, Ho Chi Minh City, Vietnam

**DOI:** 10.1186/s13104-015-1806-4

**Published:** 2015-12-22

**Authors:** Nguyen Binh Hoa, Nguyen Viet Nhung, Pham Huyen Khanh, Nguyen Viet Hai, Bui Thi Tu Quyen

**Affiliations:** National Tuberculosis Programme Vietnam, Hanoi, Vietnam; Centre for Operational Research, International Union against Tuberculosis and Lung Disease, Paris, France; Vietnam Association for Tuberculosis and Lung Disease, Hanoi, Vietnam; World Health Organization (WHO), Vietnam Country Office, Hanoi, Vietnam; Hanoi Medical University, Hanoi, Vietnam; Department of Epidemiology and Biostatistics, The Hanoi School of Public Health, Hanoi, Vietnam

**Keywords:** Vietnam, Multidrug-resistant TB, Green light committee, Adverse drug reaction

## Abstract

**Background:**

Treatment outcomes of a high proportion of inpatients with multi-drug resistant tuberculosis (MDR-TB) were not reported to the Vietnamese National Tuberculosis Program because they received treatment outside of the green light committee (GLC) program. The study aimed (1) to describe the strengths and weaknesses of treatment of GLC and non-GLC MDR-TB patients as well as the factors influencing treatment completion and (2) to determine the incidence of adverse drug reactions.

**Results:**

This cross-sectional study comprised two elements: (1) in-depth interviews with clinical doctors, hospital pharmacists; and focus group discussions with MDR-TB patients; and (2) a review of the charts of all GLC and non-GLC MDR-TB patients in 2010. A total of 282 MDR-TB patients were recruited, including 79(28 %) MDR-TB patients treated through the GLC program and 203(72 %) MDR-TB patients treated outside of the GLC program. The main strengths of GLC treatment were the supply of quality assured second line TB drugs, routine monitoring and clinical evaluation, free diagnostic tests and close clinical monitoring. The greatest barriers to patients treated outside of the GLC program was difficulty paying for second line TB drugs and other treatment costs. There was no significant difference between the incidence of adverse events among GLC (46.8 %) and non-GLC treated patients (52.2 %; p = 0.417). Among 143 patients who reported 226 adverse reaction events, arthralgia/joint pain (35.8 %), gastrointestinal (14.2 %), ototoxicity (8.4 %), cutaneous (6.6 %), and giddiness (5.8 %) were the most common.

**Conclusions:**

The non-GLC MDR-TB patients face substantial barriers to treatment, and require greater support if they are to complete treatment and improve disease outcomes. Staff training about the management of adverse drug reactions is needed.

## Background

Vietnam ranks 12th among the 22 high-TB burden countries, and is one of the 27 high-multi-drug resistant tuberculosis (MDR-TB) burden countries in the world. In 2013, the estimated incidence of TB all forms was 144/100,000 population, and prevalence was 209/100,000 population and the estimated TB all forms case detection rate was 76 % [1].

The most recent national drug resistant survey (DRS) in 2011 estimated the prevalence of MDR-TB to be 4 % among new patients and 23 % among the previously treated cases, which translates to an estimated number of 5100 MDR-TB cases per year (3000 new and 2100 re-treatment cases) [1].

The World Health Organization (WHO) and the Stop TB Partnership have been supporting countries to manage multidrug-resistant tuberculosis (MDR-TB) through the green light committee (GLC) Initiative, providing access to quality-assured second-line anti-TB medications under programmatic management conditions [2]. According to the programmatic management of drug-resistant TB (PMDT) monitoring report in March 2012, first-line (FLD) and second-line anti-TB drugs (SLDs) of unknown quality are still available in Vietnam. The SLDs still are available for purchase in pharmacies and chemist shops in local include Kanamycin (Km); Levofloxacin (Lfx); Ethionamide (Eto), Cycloserine (Cs) and p-aminosalicylic acid (PAS). Many non-GLC drugs are imported without being subjected to defined quality assurance processes. Availability of potentially sub-standard products to creates the potential for inadequate therapies, and an increase in the incidence of extensively drug resistant (XDR) TB [3].

In Vietnam, two evaluations of PMDT in February 2011, and March 2012 revealed that a large proportion of MDR-TB patients treated within public lung disease hospitals were receiving non-GLC drugs, and that their treatment outcomes were often not reported to the national tuberculosis programme (NTP) [3]. The second evaluation found that in some facilities, MDR patients were managed by doctors who had inadequate training for PMDT. It also showed that prescribed and self-administered SLD drugs of undetermined quality were being given for prolonged periods. Bacteriologic outcomes and documentation of compliance was limited, meaning that outcomes could not be evaluated. The report concluded that unregulated use of non-standardised drugs and an absence of appropriate quality assurance processes outside of the Global Fund administered programs may compromise patient outcomes [3].

In order to better understand about MDR-TB treatment in public TB/MDR-TB facilities, we conducted a study to characterise the perceived strengths and weaknesses of treatment for MDR-TB within and outside of the GLC program at the largest public TB hospital dealing with MDR-TB patients in Ho Chi Minh City (HCMC), Vietnam. We also evaluated the incidence of adverse drug reactions in this population.

## Methods

### Study design

This cross-sectional study was carried out in Pham Ngoc Thach hospital (PNT), HCMC, Vietnam in 2012; including quantitative and qualitative components.

### Study participants and sampling

Qualitative component: We conducted four In-depth interviews with three MDR-clinical doctors and a pharmacist in PNT. Two groups of MDR-TB patients, both GLC-approved and non-GLC, have been enrolled in this study based on the following criteria: (1) they had registered for MDR-TB treatment during the years 2010–2012; (2) they live near the study hospitals, or are still hospitalized for MDR treatment. There were ten patients in each group.

Quantitative component: We also extracted information of treatment and adverse events among 282 GLC and non-GLC MDR-TB patients enrolled for treatment in 2010, based upon chart review. 79 of them (28 %) belong to the GLC MDR-TB patient group and 203 (72 %) of them belong to the non-GLC MDR-TB patient group.

### Measurement

In this study, we collected data/information from three sources: (1) face to face interviews with health staffs (2) focus group discussions (FGD) with MDR-TB patients and (3) review of patient medical records.

Variables/themes

- Variables evaluated in the quantitative component: The main variable was adverse drug reactions during treatment. Information relating to the treatment of MDR-TB patients: GLC or non-GLC treatment; treatment default; and the MDR-TB treatment regimen. We also extracted patients’ gender and age from MDR-TB patient charts.
- Themes from the qualitative component: The strengths and weakness of GLC treatment; the barriers to patients treated outside of GLC program; and reasons for patients’ choice of either GLC or non-GLC treatment.

### Data collection

Data were collected by a survey team of five members from the NTP staff using a check list and semi-structured questionnaires. The team was trained to collect data from treatment cards and patients’ charts for all MDR-TB patients treated in 2010. In-depth interviews and FGDs were undertaken by the principal investigator. Data were collected during December 2012.

### Data entry and analysis

Qualitative data: Data analysis has been done thematically using open coding. Information has been summarized and presented by theme/subthemes.

Quantitative data: Data were entered in an electronic data file using EpiData Entry software (http://www.epidata.dk) and analyzed using Stata version 10. Hypothesis testing was performed using the Chi square tests for the difference in proportions with the alpha value of 0.05.

### Ethical issues

Ethical approval was obtained from the Ethical Committee of the Vietnam National Lung Hospital. Verbal consent was obtained from all persons taking part in the interview. Information gathered from clinicians, pharmacists and patient data were kept confidential. Patients’ names were not collected.

## Results

A total of 282 MDR-TB patients enrolled during 2010 at PNT hospital were recruited from patient’s charts. Of those 79 (28 %) patients receiving treatment with GLC drugs and 203 (72 %) receiving non-GLC MDR-TB drugs. Of 282 MDR-TB patients, 182 (65 %) were males, and there was no significant difference in the sex distribution between the GLC and non-GLC groups (Table 1). The majority of MDR-TB patients belonged to the 30–45 age group (58 % in GLC group and 60 % in non-GLC group). The mean age of 79 GLC patients was 44.3 years old (SD = 12.8) and that of 203 non-GLC patients was 40.4 (SD = 13.2).

**Table 1:** General and treatment characteristics of MDR-TB patients in GLC and non-GLC groups, in Pham Ngoc Thach hospital, 2010

Characteristics	GLC (n = 79)		Non-GLC (n = 203)		p value
	n	%	n	%	
Sex					
Male	57	72.2	125	61.6	0.226
Female	21	26.6	76	37.4	
Unknown	1	1.3	2	1.0	
Age groups					
<30	16	20.3	49	24.1	
30–45	46	58.2	122	60.1	0.302
>45	16	20.3	32	15.8	
No information	1	1.3		0.0	
Treatment regimen					
Regimen 4A	43	54.4	1	0.5	
Regimen 4B	34	43.0	0	0.0	
Others	1	1.3	202	99.5	
No information	1	1.3	0	0.0	
Treatment outcome					
Cured	62	78.5	99	48.8	<0.001
Completed	5	6.3	10	4.9	
Failure	2	2.5	5	2.5	
Default	5	6.3	52	25.6	<0.001
Died	3	3.8	0	0	
Transfer-out	0	0	2	1	
Still on treatment	0	0	1	0.5	
Not evaluated	2	2.5	34	16.8	

Table 1 shows some of the regimens most frequently used in the treatment of non-GLC patients. In general, the non-GLC patients did not follow regimens 4A and 4B stated in the Vietnamese national guidelines. Ethionamide was used instead of Prothionamide in the regimen; hence, this regimen differs from regimens 4A or 4B.

### Reasons for choosing non-GLC treatment

All of three clinical doctors reported that the main reasons that patients choose the non-GLC treatment were that (a) they did not meet criteria for enrolment into the GLC program, such as that they were not long-term residents of HCMC or (b) patients did not accept treatment as inpatients.

### Attitudes among MDR-TB patients towards non-GLC and GLC treatments

Among ten MDR-TB patients receiving GLC drugs that took part in FGD, seven perceived those drugs to have good quality. All of them said that they would receive free testing with sputum smear, CXR and culture; eight of them perceived they would be monitored closely by health staff within official DOTS treatment centres. GLC patients had lower default rates than did non-GLC MDR patients (5 defaulters out of 79 GLC patients (6.3 %) vs. 52 defaulter out of 203 non-GLC patients (25.6 %, p < 0.001) Table 1.

Among the group of ten MDR-TB patients who did not receive GLC drugs, eight of them made their choice because they did not want to travel to health care facilities each day to receive treatment. They were also were able to work during treatment (six of them were still working during treatment). This was particularly important to patients who working far from home, and wealthier patients who did not want to be identified with other MDR-TB patients in their neighborhood. In contrast, patients receiving GLC treatment needed to attend TB daily for 18–24 months so that they could receive injectable antibiotics or oral SLDs. This presented a substantial challenge to MDR-TB patients with severe disease, or those who were the primary income earners and worked far from home.

The greatest challenge for non-GLC MDR-TB patients was their ability to pay for SLDs and other expenses, all of ten non-GLC MDR-TB patients mentioned about it. Both health staff and patients reported that the costs of meals, transportation and follow-up tests were substantial over the long treatment periods (normally 18–24 months). Moreover, as patients self-administered their treatment, important ADRs were unlikely to be resolved in a timely and safe manner. These factors are likely to contribute to the higher default rate seen among non-GLC MDR-TB patients.

### Factors influencing treatment completion by MDR patients

The factors associated with not completing treatment among GLC patients included: limited financial resources, employment status (i.e., patients have to work for living) and the perception that free drugs were of a lower quality. Factors that encouraged compliance with treatment among GLC patients included provision of the drugs free of charge, supervision of therapy, the use of treatment supporters (a commune health worker and family member), provision of ancillary therapies to treat ADRs and a budget to support patients traveling from home.

Among non-GLC patients, there was no restriction regarding the use of TB drugs, and doctors were unable to ensure compliance (i.e., if patients interrupted their treatment, doctors were only able to give advice). Other reasons for non-compliance included the long duration of treatment, such that when patients felt better they believed that they had been cured and could stop treatment.

The non-GLC MDR TB patients have to pay all treatment costs themselves, including expensive SDL. If the patients had sufficient resources to afford treatment and SLD, they were likely to take the drugs according to medical advice. Others factor associated with adherence or treatment completion among non-GLC patients were their economic status and the presence of strong domestic support for treatment, such as strong family support.

### The challenges patients faced during MDR-TB treatment

Among patients receiving non-GLC treatment, doctors prescribed the SLD regimen for 1 month. Treated patients needed to purchase the drugs at the hospital pharmacy. Patients were requested to self-administer the drugs, and to return to the hospital monthly for a clinical check-up, sputum smear and culture. Furthermore, such patients were requested to attend to have a chest X-ray every 2–3 months.

Non-GLC patients felt most secure receiving treatment in the hospital, and accepted the specialist knowledge of the doctors. The main reason that patients choose treatment with non-GLC regimens was that they felt they had no choice, as they did not meet the required criteria for free treatment.

### The adverse drugs reaction

Table 2 presents the cumulative incidence of adverse drugs events occurring during treatment of GLC and non-GLC MDR-TB patients in PNT hospital in 2010. There was no significant difference between the incidence of adverse drug events during treatment between GLC and non-GLC MDR-TB patients (46.8 vs. 52.2 %, p = 0.417).

**Table 2:** Adverse drug events occurred during treatment of GLC and non-GLC MDR-TB patients, Pham Ngoc Thach hospital, 2010

Adverse drugs event	GLC		Non-GLC		p value
	n	%	N	%	
Total	79	100.0	203	100.0	0.417
No adverse events	42	53.2	97	47.8	
Adverse events	37	46.8	106	52.2	0.779
1 event	25	31.6	63	31.0	
2 events	9	11.4	27	13.3	
≥3 events	3	3.8	16	7.9	

Among 143 patients who reported 226 adverse reaction events, the most common adverse event was arthralgia/joint pain (35.8 %), followed by gastrointestinal symptoms (14.2 %), ototoxicity (8.4 %), cutaneous reactions (6.6 %), and dizziness (5.8 %). Among these 226 adverse reaction events, 25 were classified as severe, and led to a change in regimen or complete cessation of therapy (Table 3).

**Table 3:** Frequency of adverse drug events observed during MDR treatment, Pham Ngoc Thach hospital, 2010

Type of adverse event^a^	Total		Mild^b^	Moderate^c^	Severe^d^	No information
	n	%				
Total	226	100.0	78	32	25	91
Arthralgia/joint pain	81	35.8	25	16	7	33
Gastrointestinal	32	14.2	18	2	0	12
Ototoxicity	19	8.4	8	3	4	4
Cutaneous reaction	15	6.6	4	4	0	7
Giddiness	13	5.8	2	1	0	10
Psychiatric symptoms	10	4.4	5	1	3	1
Hepatitis	7	3.1	1	2	4	0
Peripheral neuropathy	7	3.1	3	1	0	3
Renal toxicity	5	2.2	2	0	3	0
Visual symptoms	5	2.2	4	0	0	1
Insomnia	5	2.2	0	0	0	5
Hypothyroidism	3	1.3	0	2	1	0
Central nervous system	1	0.4	0	0	0	1
Others	23	10.2	6	0	3	14

^a^ An individual patient could have had more than 1 adverse event during the MDR treatment period

^b^ Mild: the drug was continued at the same dose, with or without ancillary drug prescribed, e.g., reassurance

^c^ Moderate: drug was continued at a reduced dose, with an ancillary drug prescribed

^d^ Severe: drug was changed or stopped and/or all medical treatment was stopped

## Discussion

The most common reason given by patients for choosing non-GLC treatment was that they did not satisfy inclusion criteria for enrolment into GLC program. This was often because they were not resident in HCMC, or had difficulty in receiving treatment as inpatients. At the time of this study (2010), enrolment in the subsidized GLC treatment program required that patients were diagnosed at PNT hospital or had been referred from one of the HCMC district TB units, and that they were official residents of HCMC. If they met these criteria, they could receive 1–2 months of free inpatient therapy during their intensive phase, and subsequent supervised treatment at district TB units. Many of these patients were resident outside of HCMC, or were required to attend local district TB units to receive supervised daily SLD therapy. At that time, few other provinces in Vietnam had offered subsidized PMDT, including the major cities of HCMC, Can Tho, and Ha Noi, as well as Binh Dinh province and the northern K74 and K71 hospitals. Other provincial TB centers and district TB had not yet received training in the management of MDR-TB patients. Hence, few MDR-TB patients from outside of the selected provinces where treatment was offered could access continuous MDR-TB treatment after their initial inpatient therapy. After the year 2011, currently, the NTP has been extended PMDT to 10 treatment and 35 satellite sites in 45 provinces. Since that time, a growing number of patients with MDR-TB patients have been able to satisfy the criteria for subsidized MDR therapy.

The most substantial part of MDR-TB treatment is the cost of drugs. Expensive drugs must be taken for a long duration (18 months), and ongoing laboratory tests, travel expenses, examination fees and other costs are beyond the financial means of many patients. This likely explains much of the high drop-out rate from non-GLC therapy. In the future, drug costs and additional financial supplements will be required to reduce the default rate and reduce the substantial difference in treatment completion between GLC and non-GLC MDR-TB patients.

The official guidelines for MDR-TB treatment by the Vietnam NTP at the time of the study was a 4A regimen for new cases [6 *Z E Km Lfx Pto Cs* (*PAS*)/12 *Z E Lfx Pto Cs* (*PAS*)] and a 4B regimen for retreated cases [6 *Z E Cm Lfx Pto Cs* (*PAS*)/12 *Z E Lfx Pto Cs* (*PAS*)] [4]. Many non-GLC patients, received regimens 4A or 4B, except Prothionamide was replaced by Ethionamide (Table 1). One possible explanation for this difference is that Prothinamide is more difficult to obtain in private pharmacies than Ethionamide, and that the efficiency of the two drugs is comparable. Most treatment regimens contained five drugs in the intensive phase, with the injectable drug being ceased in the continuous phase. Among the five drugs used in the intensive phase, the regimen always included injectable drugs (most often Amikacin/Kanamycin, with a few regimens including Capreomycin instead). One drug belong to fluoroquinolone group; (in decreasing frequency of used: Ofloxacin/Levofloxacin/Moxifloxacin/Gatifloxacin/Ciprofloxacin). Most regimen included Ethionamide. The fourth drug was either cycloserine or PAS, in order of decreasing frequency of use. Many regimens used both Cycloserine and PAS. And the last drug included in standard regimens was the first line drugs (Pyrazinamide/Ethambutol).

In this study, 47 % of patients taking GLC drugs reported adverse reactions and 52 % of non-GLC patients reported adverse reactions. However, the lower compliance in non-GLC patients (or higher of defaulter rate) may have reduced the likelihood of documented adverse events in this group. This was consistent with the opinion of doctors, that there was no significant difference in the rate of adverse reactions between GLC patients and non-GLC patients.

The frequency of adverse reactions in this study was quite low in comparison to other studies. Among 2027 MDR-TB patients enrolled from 2000 to 2004 in Latvia, 807 (79 %) experienced at least one adverse event [5]. In study in Tomsk, Russia, of 244 MDR-TB patients enrolled from September 2000 to 2002, 73 % had experienced at least one adverse event [6]. A study of 38 MDR-TB patients in India in the years 2006–2007 showed that 58 % patients reported adverse drug reactions which required dose reduction or termination of the offending drug [7]. Another study was collected data on adverse events from five DOTS-Plus sites in Estonia, Latvia, Peru (Lima), the Philippines (Manila) and the Russian Federation (Tomsk Oblast), the results showed that among 818 MDR-TB patients, only 2 % of patients stopped treatment, but 30 % required removal of the suspected drugs from the regimen due to adverse events [8].

The most common adverse drug reactions were also observed varies between published studies. In Nathanson et al., the five most common adverse events were nausea/vomiting (33 %), diarrhea (21 %), arthralgia (16 %), dizziness/vertigo (14 %) and hearing disturbances (12 %) [8] [5]. In another Latvian study, the mostly commonly reported event were nausea (58 %), vomiting (39 %) and abdominal pain (24 %) [5]. Another study in Turkey review of the medical records of 263 MDR-TB patients reported that 69 % had side effects, most frequently ototoxicity (42 %); psychiatric disorders (21 %); arthralgia (11 %) and epileptic seizures (10 %) [9]. In our study, the most common adverse drug reactions were arthralgia or joint pain (36 %), followed by gastrointestinal disturbance (14 %).

An important implication of this study is the importance of ensuring that staff within the NTP are trained to recognize and manage common adverse events associated with use of SLDs.

To our knowledge, this is the first study to describe the incidence of adverse drug reactions among MDR-TB patients in Vietnam. Our study has limitations. Firstly, this is retrospective study, based on information in the patient’s files of the cohort of GLC and non-GLC patients treated in PNT hospitals in the year 2010, so that we only have information if it available in patient files. Secondly, the study did not use standardized definitions of adverse events, their diagnosis or the severity, this may lead to the frequency and severity of events being reported in a variable fashion. Lastly, since the program’s rules forced certain patients to choose the non-GLC option (those who lived outside HCMC or could not afford to be admitted to the hospital), the comparison in this study is not really an evaluation of GLC drugs or the GLC program, but a comparison between two different groups of patients.

## Conclusions

In conclusion, there are many perceived disadvantages of taking non-GLC treatment among patients attending PNT hospital in HCHC, Vietnam. These patients require greater support from the NTP to complete MDR treatment and improve their treatment outcomes. Training on management of adverse drug reactions for health staff is also needed.

## Abbreviations

CXR: chest X-ray
FGD: focus group discussion
FLDs: first-line anti tuberculosis drugs
GLC: green light committee
HCMC: Ho Chi Minh city
MDR-TB: multidrug-resistant
NTP: national tuberculosis programme
PMDT: programmatic management of drug-resistant tuberculosis
PNT: Pham Ngoc Thach hostital
SLDs: second-line anti tuberculosis drugs
WHO: World Health Organization
XDR: extensively drug resistant
